# Long-Term Mortality in Cardioinhibitory Carotid Sinus Hypersensitivity Patient Cohort

**DOI:** 10.36660/abc.20190008

**Published:** 2020-02

**Authors:** Gustavo de Castro Lacerda, Andrea Rocha de Lorenzo, Bernardo Rangel Tura, Marcela Cedenilla dos Santos, Artur Eduardo Cotrim Guimarães, Renato Côrtes de Lacerda, Roberto Coury Pedrosa

**Affiliations:** 1 Instituto Nacional de Cardiologia, Rio de Janeiro, RJ - Brazil; 2 Universidade Federal do Rio de Janeiro, Rio de Janeiro, RJ - Brazil; 3 Hospital Federal de Bonsucesso, Rio de Janeiro, RJ - Brazil

**Keywords:** Carotid Sinus,Massage/mortality, Bradycardia, Syncope, Cardiac Pacing, Artificial

## Abstract

**Background:**

Cardioinhibitory carotid sinus hypersensitivity (CICSH) is defined as ventricular asystole ≥ 3 seconds in response to 5-10 seconds of carotid sinus massage (CSM). There is a common concern that a prolonged asystole episode could lead to death directly from bradycardia or as a consequence of serious trauma, brain injury or pause-dependent ventricular arrhythmias.

**Objective:**

To describe total mortality, cardiovascular mortality and trauma-related mortality of a cohort of CICSH patients, and to compare those mortalities with those found in a non-CICSH patient cohort.

**Methods:**

In 2006, 502 patients ≥ 50 years of age were submitted to CSM. Fifty-two patients (10,4%) were identified with CICSH. Survival of this cohort was compared with that of another cohort of 408 non-CICSH patients using Kaplan-Meier curves. Cox regression was used to examine the relation between CICSH and mortality. The level of statistical significance was set at 0.05.

**Results:**

After a maximum follow-up of 11.6 years, 29 of the 52 CICSH patients (55.8%) were dead. Cardiovascular mortality, trauma-related mortality and the total mortality rate of this population were not statistically different from that found in 408 patients without CICSH. (Total mortality of CICSH patients 55.8% vs. 49,3% of non-CICSH patients; p: 0.38).

**Conclusion:**

At the end of follow-up, the 52 CICSH patient cohort had total mortality, cardiovascular mortality and trauma-related mortality similar to that found in 408 patients without CICSH.

## Introduction

Carotid sinus hypersensitivity (CSH) is characterized by ventricular asystole ≥3 seconds, known as cardioinhibitory carotid sinus hypersensitivity (CICSH) or systolic blood pressure fall ≥50 mmHg (vasodepressor carotid sinus hypersensitivity) in response to 5-10 seconds of carotid sinus massage (CSM).^1,2^ Epidemiologic studies of patients >40 years old have shown that this population have a high prevalence of CSH (10-50%).3,4 This prevalence is even higher among men and in patients with atherosclerosis.^3,4^

Carotid sinus hypersensitivity can be present with or without spontaneous symptoms.^1^ On the other hand, diagnosis of carotid sinus syncope (CSS) requires the presence of vasodepressor or CICSH and syncope.1,5 Carotid sinus syncope is considered one of the most frequent causes of syncope in the elderly.^6^ Treatment is generally indicated for CSS patients to reduce recurrence of symptoms.^1,2^ The concern that a prolonged asystole episode could lead to serious trauma, brain injury, pause-dependent ventricular arrhythmias and death is also used to justify treatment.^5,7^ The main objective of present study is to describe the long-term mortality rate of a cohort of CICSH patients. Secondly, it compares total mortality, cardiovascular mortality, mortality due to ischemic heart disease and trauma-related mortality of this patient cohort with that of a cohort of patients without CICSH.

## Methods

In 2006, in the first phase of the present study, 502 patients were randomly selected among 1,686 outpatients ≥50 years of age referred to electrocardiography in a public general hospital in Rio de Janeiro, Brazil. These 502 patients were submitted to CSM, 52 (10,4%) were identified with CICSH (ventricular asystole ≥3) and, in 450, cardioinhibitory reflex was absent. In all cases, CSM was performed in the supine position, initially on the right side, then on the left side for 10 seconds by a single investigator. More patient selection details and more information about CSM can be found in a previous article.^8^

In the present phase of the study, the 502 patients submitted to CSM in 2006 were divided into groups. The first group was formed by the 52 CICSH patients and, for comparison purposes, a second group of 450 patients without CICSH was studied. Survival data was assessed through active follow-up and review of Rio de Janeiro deaths database and the Rio de Janeiro medical admissions database. In the latter, we have searched for all patients who had permanent a pacemaker paid by the state government of Rio de Janeiro. In all cases, we have considered the cause of death described in Rio de Janeiro deaths database. Cardiovascular deaths were those registered under chapter IX of the International Statistical Classification of Diseases and Related Health Problems 10th Revision (ICD-10); ischemic heart disease deaths were those registered under ICD-10 codes I20 - I25, and trauma related deaths were those registered under ICD codes S00 - T14, T66 - T98, V01 - V29, V80 - V94, V98 - W19, W65 - W74, Y85 - Y89.

### Ethical approval

The protocol was approved by the local ethics committee (approval statement number 2.383.341) conforming to the standards of the Brazilian National Committee of Research Ethics (resolution 466/2012).

### Statistical analysis

All data were analyzed using the R Core Team (2018) software. The Shapiro-Wilk test was used to verify the normality of the data. Normally distributed continuous data are shown as mean and standard deviation and the differences between the two groups are compared using unpaired Student's t-test. Categorical data are presented as absolute and relative frequencies and are compared using x2 or Fisher's exact tests as appropriate. The level of statistical significance was set at 0.05.

Time to event was defined as the time between the date of CSM and death or end of the study; December 31, 2017. The time to event was analyzed using the Kaplan-Meier survival curves, which were compared using the log-rank test. Risk factors associated with mortality were analyzed using the Cox regression analysis. Two models were created, the first adjusted by sex, age and presence of atherosclerosis; the second model made additional adjustments for smoking history, history of hypertension, diabetes and dyslipidemia.

## Results

### Patients’ characteristics

In the first phase of the study, 52 CICSH patients were identified among the 502 patients submitted to CSM.^8^ Only 7 of the 52 CICSH patients had a history of syncope and 40 of them used negative chronotropic drugs. Those 52 patients were advised to avoid inadvertent stimulation of the carotid sinus and, in 12, the dosage of negative chronotropic drugs was reduced. At that time, none of the 52 patients has been submitted to permanent pacemaker implantation.

The baseline characteristics of the patients with and without CICSH are presented in table 1. Patients with CICSH were more likely to be male and had higher prevalence of structural heart disease and atherosclerosis.

**Table 1:** Baseline characteristics of the patients with and without CICSH

	42 patients lost to follow-up (without CICSH)	408 patients without CICSH	52 CICSH patients	52 CICSH x 408 without CICSH P value. OR (95% CI)
Male sex	14/42 (33.3%)	206/408 (50.5%)	39/52 (75.0%)	0.001 OR: 2.94 (1.52-5.67)
Age (mean ± SD)	65.4 ± 10.4	64.93 ± 9.74	66.31 ± 8.15	0.33
Age ≥ 65 years	20/42 (47.6%)	203/408 (49.8%)	31/52 (59.6%)	0.18
Heart rate before CSM (mean ± SD)	68.6 ± 13.6	68.7 ± 14.19	62.4 ± 15.6	0.003
Unexplained falls or syncope in the year preceding CSM	8/42 (19.0%)	56/408 (13.7%)	7/52 (13.5%)	0.95
Structural heart disease	19/42 (45.2%)	277/408 (67.9%)	46/52 (88.5%)	0.002 OR: 3.62 (1.51-8.70)
Atherosclerosis	18/42 (42.8%)	198/408 (48.5%)	37/52 (71.2%)	0.002 OR: 2.61 (1.39-4.91)
History of AMI	10/42 (23.8%)	128/408 (31.4%)	28/52 (53.8%)	0.001 OR: 2.55 (1.42-4.58)
Previous myocardial revascularization	5/42 (11.9%)	88/408 (21.6%)	20/52 (38.5%)	0.007 OR: 2.27 (1.23-4.17)
Previous CABG	2/42 (4.8%)	58/408 (14.2%)	16/52 (30.8%)	0.002 OR: 2.68 (1.40-5.14)
Previous PCI	3/42 (7.1%)	30/408 (7.4%)	4/52 (7.7%)	0.93
Atrial fibrillation	2/42 (4.8%)	20/408 (4.9%)	2/52 (3.8%)	0.73
Normal ECG	13/42 (31%)	112/408 (27.5%)	8/52 (15.4%)	0.06
Negative chronotropic drug use	28/42 (66.6%)	235/408 (57.6%)	40/52 (76.9%)	0.007 OR: 2.45 (1.25-4.18)
Hypertension	29/42 (23.8%)	311/408 (76.2%)	40/52 (76.9%)	0.91
Diabetes	10/42 (26.2%)	93/408 (22.8%)	14/52 (26.9%)	0.51
Dyslipidemia	20/42 (47.6%)	215/408 (52.7%)	35/52 (67.3%)	0.046 OR: 1.84 (1.00-3.40)
Smoking	7/42 (16.7%)	41/408 (10%)	10/52 (19.2%)	0.047 OR: 2.13 (0.99-4.56)

CICSH: cardioinhibitory carotid sinus hypersensitivity; OR: Odds ratio; CSM: carotid sinus massage; AMI: acute myocardial infarction; CABG: coronary artery bypass grafting; PCI: Percutaneous coronary artery intervention.

### Follow-up of the 52 CICSH patients

Twenty-seven of the 52 CICSH patients were actively followed up. At the end of the study, none of them had been submitted to permanent pacemaker implantation, 19 were alive and 8 had died. Data about the remaining 25 patients were retrieved at Rio de Janeiro databases of death and medical admissions. Twenty-one of those were dead and 4 were alive. None of those patients had been submitted to permanent pacemaker implantation.

**Figure 1:**
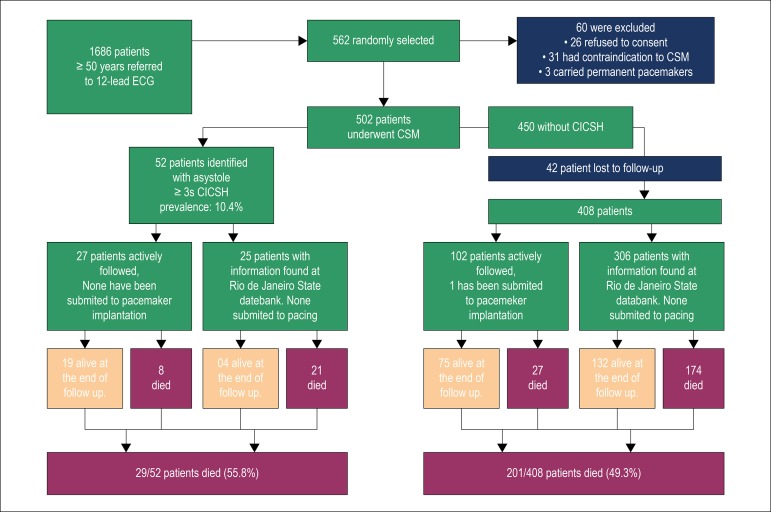
Study design and results. CSM: Carotid sinus massage; CICSH: cardioinhibitory carotid sinus hypersensitivity.

Overall, 29 of the 52 patients (55.8%) identified with CICSH had died at the end of the study (maximum follow up time of 11,6 years). Figure 1

Furthermore, the mortality rate of the 7 CICSH patients with history of syncope was 57,1%. This mortality rate was similar to that found in the 45 CICSH patients that did not have this symptom (55,5%).

### Follow-up of patients without CICSH

We could not find any information in 42 of the 450 patients without CICSH. One hundred and two patients were actively followed up. Data about the remaining 306 patients without CICSH were retrieved at Rio de Janeiro databases of death and medical admissions. Overall, 201 of the 408 patients without CICSH were dead (49.3%) at the end of follow-up, none had been submitted to permanent pacemaker implantation. One of the 207 patients that was alive at the end of follow-up had been submitted to permanent pacemaker implantation due do complete AV block.

### Patients with and without CICSH - Endpoint comparisons

Figure 1 outlines the study design and compares the deathrate of patients with and without CICSH.

**Figure 2:**
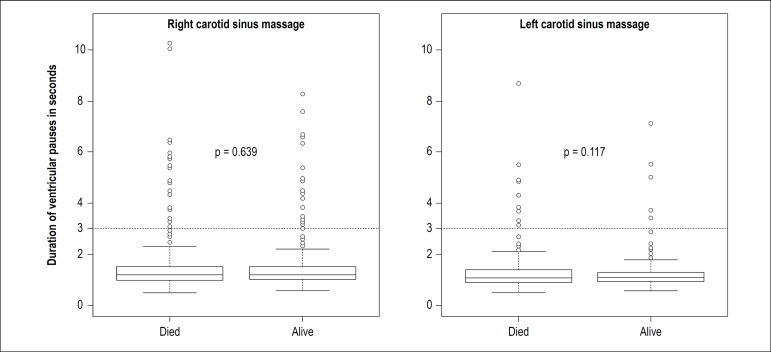
Duration of the longest RR interval observed during right and left carotid sinus massage. Boxplots on the left of each square represent patients who died during follow-up. Boxplots on the right represent patients who were alive at the end of the study.

Figure 2 shows the distribution of responses to right and left CSM in patients who died during follow-up and in patients who were alive at the end of the study. Median duration of RR intervals observed during CSM were similar in both groups of patients.

**Figure 3:**
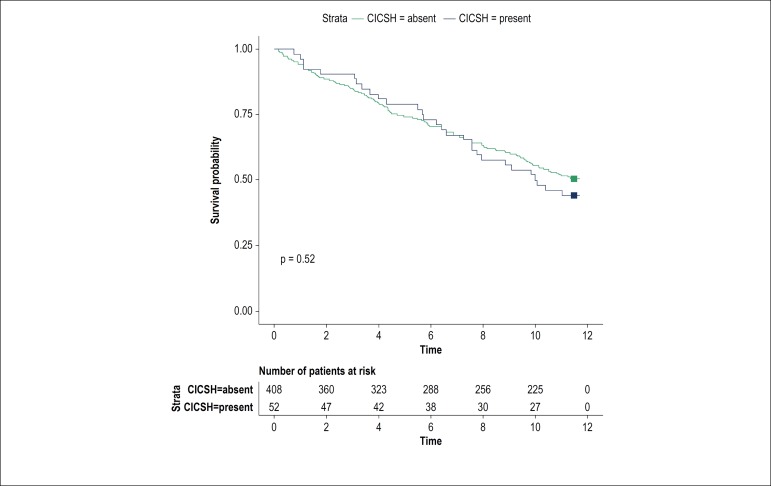
Survival curves of patients with (in blue) and without CICSH (in red) CICSH: Cardioinhibitory carotid sinus hypersensitivity.

Table 2 compares the total mortality, cardiovascular mortality, mortality due to ischemic heart disease and trauma-related mortality of the 52 CICSH patients with the 408 patients without CICSH. Survival curves are presented in figure 3. The total mortality rate of the 52 CICSH patients was 21.1% at 5 years and 51.9% at 10 years, with median survival time of 10.0 years (95% CI: 7.4 - 12.6 years). The survival curves of patients with and without CICSH were similar without any significant statistical difference. Both Cox regression models failed to reveal any association between CICSH and mortality. In both models, age at the time of CSM, and presence of atherosclerosis were independently associated with mortality. (Table 3)

**Table 2:** Mortality at the end of follow-up of patients with and without CICSH

	With CICSH	Without CICSH	p value
Number of dead patients at the end of follow-up	29/52 (55.8%)	201/408 (49.3%)	0.38
Number of cardiovascular deaths	11/52 (21.2%)	76/408 (18.6%)	0.66
Number of coronary artery disease related deaths	7/52 (13.5%)	32/408 (7.8%)	0.17
Number of cerebrovascular related deaths	2/52 (3.8%)	13/408 (3.2%)	0.80

CICSH: Cardioinhibitory carotid sinus hypersensitivity.

**Table 3:** Cox regression results and relation between CICSH and all-cause mortality

	Odds Ratio	95% Confidence Interval	p value
**Cox model 1**			
CICSH present	0.921	0.618 - 1.372	0.686
Age	1.037	1.022 - 1.051	< 0.001
Male sex	1.144	0.874 - 1.498	0.328
Atherosclerosis	1.733	1.321 - 2.276	< 0.001
**Cox model 2**			
CICSH present	0.946	0.633 - 1.412	0.785
Age	1.043	1.028 - 1.058	< 0.001
Male sex	1.078	0.820 - 1.418	0.588
Hypertension	1.032	0.745 - 1.431	0.847
Dyslipidemia	0.645	0.486 - 0.855	0.002
Diabetes	1.529	1.135 - 2.062	0.005
Smoking	1.617	1.090 - 2.400	0.0170
Atherosclerosis	1.884	1.408 - 2.522	< 0.001

CICSH: cardioinhibitory carotid sinus hypersensitivity.

## Discussion

This study demonstrates, for the first time out of the European continent, that the mortality rate of patients with CICSH is similar to that found in a population without CICSH. Median survival of the 52 CICSH patients was 10.0 years (95% CI: 7.4 - 12.6 years). Cardiovascular mortality and trauma-related mortality, important endpoints in patients with prolonged asystole episodes, were also similar in both cohorts. These results are analogous to that described by Hampton et al.^9^ Those authors did not find any association between the presence of CICSH and survival in a cohort of 1,504 English patients with CSH (median age 77 years, 59% female).^9^ In that cohort, the median survival of CICSH patients was 8 years (95% CI: 7.3 - 8.7 years).^9^ That survival was inferior to the one observed in the 52 CICSH patients described in the present study, but was not different to that found in English elderlies with CSH and pure vasodepressor response (median survival of 7 years; 95% CI: 6.4 - 7.4 years).^9^ In the same study, Hampton et al.9 described that the total mortality, cardiac mortality, stroke and trauma-related mortality of the CSH cohort were not different from that found in sex- and age-matched English patients without CSH.^9^

In another European study, the natural history of 262 patients with carotid sinus syncope was described by Brignolle et al.^10^ Eighty-nine patients (34%) died after 46 ± 23 months of follow-up.^10^ This high mortality rate was ascribed to the advanced age of the population and to the presence of important comorbidities.^10^ Similar finding were published by Sutton et al.,^7^ and by Claesson et al.^11^ Sutton et al.^7^ reported a 36% mortality rate during 5 years of follow-up.^7^ Claesson et al.^11^ surveyed 106 CSH patients (64 with CICSH). After a median follow-up time of 8.6 ± 2.1 years, the mortality rate of the 106 CSH patients was not significantly different from that found in 166 patients without CSH (32% x 22%; p = 0.073).^11^

Hence, until now, no one has been able to prove the presence of any independent relation between the presence of CICSH and mortality. All of these studies evaluated residents of the European Continent and, in all of them, the natural history of CICSH patients may have been altered by pacing therapy.^5,7,9,11^ In the present study, we have shown that the risk of death was related to population age, to the presence of atherosclerosis and to the presence of risk factors for atherosclerosis. These findings indicate that the presence of CICSH should be interpreted as a risk marker. This hypothesis is supported by our Cox regression results, which showed a relation between the risk of mortality and age at the time of recruitment, and a relation between mortality and the presence of atherosclerosis. Furthermore, the Cox regression results failed to demonstrate any relation between the presence of CICSH and mortality.

Patients with a significant fall in blood pressure after CSM are usually managed with general measures that aim to increase their blood volume, including elastic stockings, physical counterpressure maneuvers, discontinuation/reduction of hypotensive therapy, fludrocortisone and alpha-agonists.^1^ Patients with isolated or mixed cardioinhibitory response are usually managed with pacing when syncope is recurrent.^1,2,7^ However, many studies used to justify pacing were observational, without a control group, or were small randomized open-label trials with no treatment control arm.^10-12^ Those study results should be regarded with caution. The possibility of spontaneous remission of syncope, the difficulties to document the symptoms used as endpoints and the open-label design of these studies continue to raise doubts about their results. Analogous studies evaluated pacing indications in vasovagal syncope.^13-15^ In an early clinical trial, with an open-label design, pacing was able to reduce syncope recurrence. However, in a later double-blind clinical trial, pacing therapy was not advantageous and failed to have any benefit in reducing syncope recurrence.^16^

Questions about the efficacy of pacing are even stronger in patients with other types of reflex syncope. Those questions are addressed in 2 recent systematic reviews.^15,16^ Interestingly, in one of them an analysis of mortality is made.^16^ In this analysis, which includes 3 studies of patients with CICSH and 1 study of patients with vasovagal syncope, pacing therapy did not reduce mortality.^16^

Only 2 clinical trials evaluated CICSH patients with a double-blind design.^17,18^ The first was a double-blind crossover study^17^ that randomized 32 elderly patients with at least 3 falls attributed to the presence of CICSH. All patients received dual-chamber pacing. The mean age of the population was 77 years. Patients were followed up for 1 year (6 months with DDD pacing turned on, and 6 months without atrial or ventricular pacing).^17^ At the end of follow-up, the reduction in fall burden was similar in both groups.^17^ Those results were affected by a high attrition rate. Seven of the 32 patients did not finish the study, 4 of which died during follow-up (12.5% mortality rate).^17^ Three of these 4 deaths were sudden and occurred at home, 2 of which occurred in patients without pacing.^17^ Autopsy of these patients revealed one death resulting from ischemic stroke, and two from ischemic heart disease.^17^ The fourth patient died after colectomy done after mesenteric infarction.^18^

**Figure 4:**
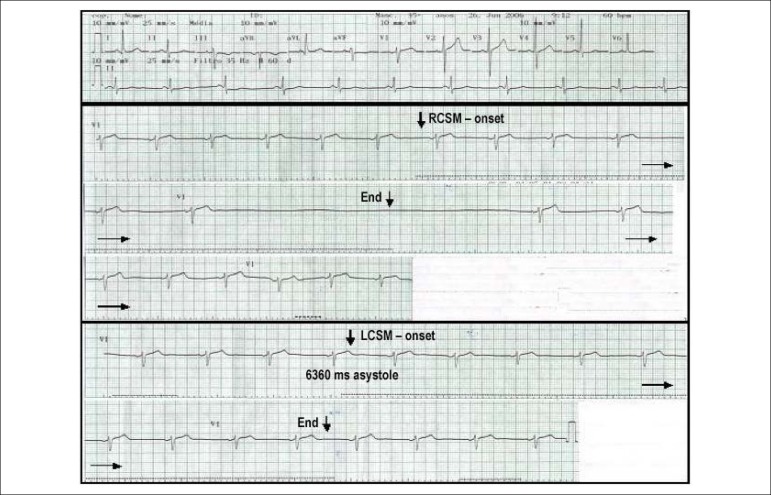
Example of a patient with CICSH. ECG of a 58 year-old male with previous percutaneous coronary intervention. He denied syncope in the past. The ECG reveals normal sinus rhythm with heart rate of 60 bpm and T-wave inversion in Lead 3 and aVF. Right carotid sinus massage triggered 6360 seconds of asystole with concomitant fall in blood pressure and pre-syncope. A few minutes later, he was submitted to left carotid sinus massage, no asystole was observed. RCSM: right carotid sinus massage.

The second clinical,^18^ trial recruited 141 elderly patients with a history of syncope or unexplained fall attributed do the presence of CICSH. Patients were randomized to dual-chamber pacing or received an implantable loop recorder. After 2 years of follow-up, fall and syncope recurrence were similar in both groups. This trial has been criticized because the larger RR interval triggered by CSM was 3.1 seconds. Hence, the magnitude of cardioinhibitory response was considered to be small. According to pathophysiological studies, cerebral ischemic anoxia reserve time is around 7 seconds in healthy military personnel,^19^ and a ventricular pause of 3 seconds is not likely to lead to loss of consciousness.^20^ So, a ventricular pause of 3 seconds is not likely to produce syncope. Based on this reasoning and based on an epidemiologic study that showed that the 95th percentile for CSM response was 7.3 seconds, Krediet et al.^20^ have proposed 6 seconds as a new cut off for the diagnosis of CICSH.^20^ In the present study, the largest RR interval triggered by CSM was 10.3 seconds, and the 95th percentile for CSM response was 4.5 seconds. Thirteen of the 502 patients submitted to CSM had an asystole episode ≥6 seconds. (Figure 4) At the end of follow-up, the mortality rate of this small group of patients was 53.8%, which is similar to the percentage found in the 447 patients followed up without a pause ≥6 seconds (53.8% vs. 49.9%; p value: 0.77).

### Study limitations

Besides reducing the heart rate and prolonging or blocking atrioventricular conduction, CSM may trigger a fall in blood pressure.^1,2^ The blood pressure fall observed after CSM is a rapid and transient phenomenon. To be properly observed, this phenomenon must be documented on a beat-by-beat basis using invasive methods or digital pletismography.^1^ Furthermore, this blood pressure fall is more commonly observed with the patient in the upright position on a tilt table.^1,6^ In 2006, in the first phase of the present study, devices used to evaluate blood pressure non-invasively on a beat-by-beat basis and tilt tables were not available in Rio de Janeiro public hospitals, so we have evaluated blood pressure response manually with a sphygmomanometer in the supine position. This method lacks sensitivity^1,6^ and, for this reason, we have decided to present only the heart rate response to CSM.

Only 7 of the 52 CICSH patients had a history of unexplained syncope, and none of them had recurrent syncope. This population had CSH, and was not affected by real CSS. It is difficult to conduct a study on the natural history of cardioinhibitory carotid sinus syncope because cardiac pacing is indicated to reduce symptoms in these patients.^1^ According to many authors, this treatment could also modify the natural history of CICSH, reducing the mortality of patients with CSS.^5,7^ As we have seen, pacing is also justified by the concern that a prolonged asystole episode could lead to serious trauma, brain injury, pause-dependent ventricular arrhythmias and death.^5,7^ Our results suggest that this concern is excessive. However, we have to emphasize that in their most recent guidelines, the Brazilian Society of Cardiology and the European Society of Cardiology continue to recommend pacing for patients with CICSH and recurrent syncope.^1,2^ It must be stressed that it is very important to document the association between symptoms and bradycardia because pauses and bradycardias without clinical significance can be easily induced by CSM in elderly individuals, especially when these patients are on negative chronotropic drugs.^1,2^

## Conclusions

The present study showed that 55.8% of the CICSH patient cohort had died after a maximum follow-up of 11.6 years. This high mortality rate was similar to that found in a cohort of patients without CICSH. Cardiovascular mortality, ischemic heart disease and trauma-related mortalities were also similar in both patient cohorts.
